# Wheat flour-derived amyloid fibrils for efficient removal of organic dyes from contaminated water

**DOI:** 10.1186/s40643-024-00737-9

**Published:** 2024-02-14

**Authors:** Dan-Dan Yang, Fu-Xiang Chang, Bo-Fan Zhang, Yang-Chun Yong

**Affiliations:** 1 Biofuels Institute, School of Environment and Safety Engineering, Jiangsu University, 301 Xuefu Road, Zhenjiang, 212013 China; 2 School of Emergency Management, Jiangsu University, 301 Xuefu Road, Zhenjiang, 212013 China; 3 Jiangsu Collaborative Innovation Center of Technology and Material of Water Treatment, Suzhou University of Science and Technology, Suzhou, 215009 China

**Keywords:** Wheat flour, Amyloid fibrils, Adsorption, Congo red, Eosin Y

## Abstract

Amyloid fibrils derived from different proteins have been proved as a promising material for adsorption of various pollutants from wastewater, which showed advantages of low cost and eco-friendliness. However, most of the amyloid fibrils derived from animal-based proteins with high environmental footprint, while more sustainable amyloid fibrils derived from plant materials are desirable. In this study, a plant-derived amyloid fibril was extracted from the commonly used wheat flour with a simple and scalable protein purification and fibrillization process. Interestingly, the amyloid fibrils showed good adsorption capacity towards typical organic dyes (Eosin Y (EY) and Congo red (CR)) from contaminated water. Adsorption kinetic analysis indicated the adsorption process to EY or CR by wheat flour amyloid well fitted with a pseudo-second-order model. The adsorption also followed a Langmuir isothermal model with adsorption capacities of 333 mg/g and 138 mg/g towards CR and EY, respectively. This work demonstrated the feasibility to utilize the plant-based amyloid fibril for organic dyes removal from contaminated water, which provided an affordable, sustainable and scalable tool for organic dyes removal from wastewater.

**Graphical Abstract:**

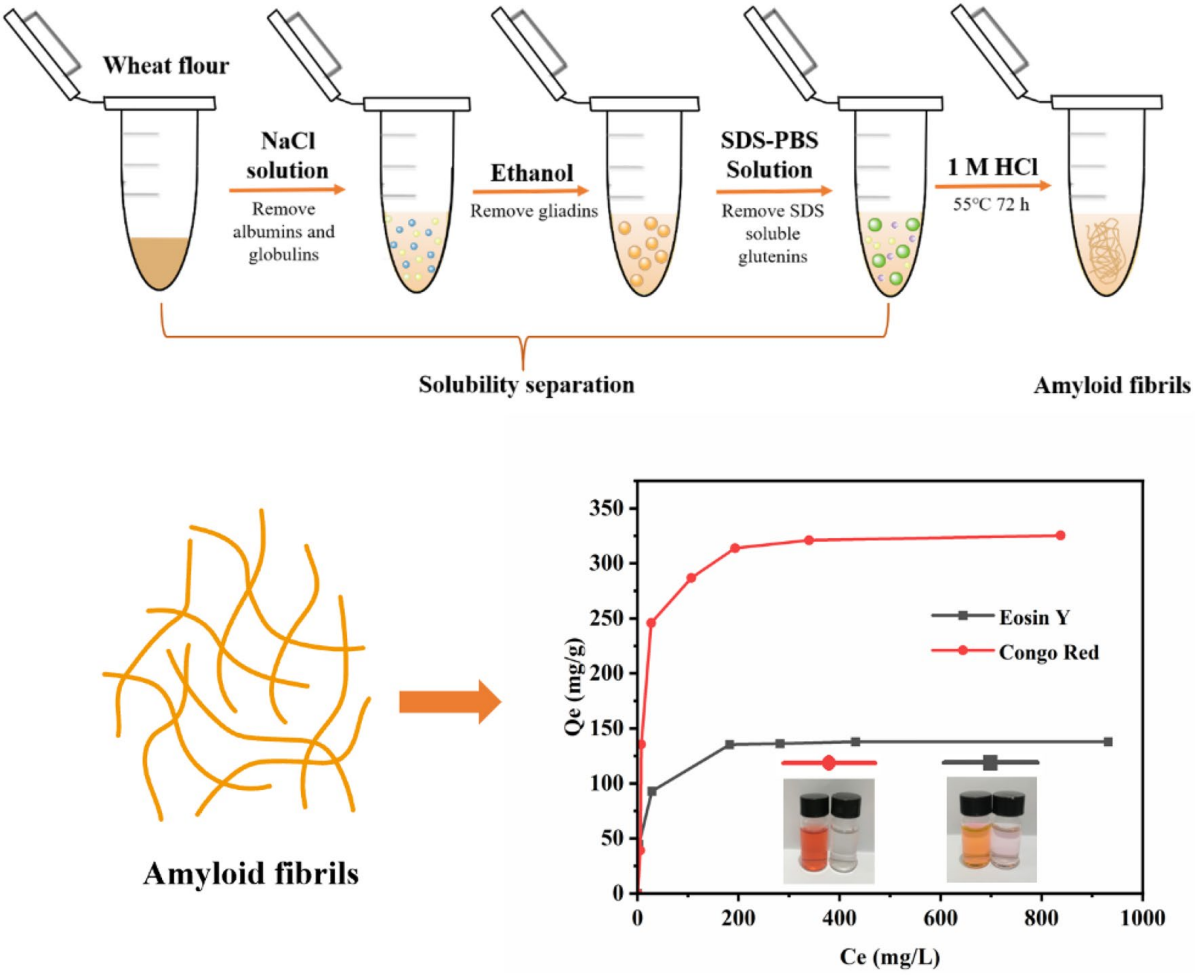

**Supplementary Information:**

The online version contains supplementary material available at 10.1186/s40643-024-00737-9.

## Highlights

- The use of plant-based amyloid fibrils for organic dyes adsorption is demonstrated.
- The wheat flour-derived amyloid fibrils exhibit excellent adsorption performance.
- The adsorption kinetics and isotherms mechanisms are explored.
- The plant-based amyloid fibrils provide sustainable tool for pollutant management.

**Supplementary Information:**

The online version contains supplementary material available at 10.1186/s40643-024-00737-9.

## Introduction

With the development of modern industry, water crisis resulted from the industrial wastewater pollution has attracted attention globally. In particularly, organic dyes that widely used in food, leather, paper and textile industries, resulting in serious wastewater pollution (Hu et al. 2006). Statistically, the global production of dyes exceeds 100,000 species with an annual production of 7 × 10^5^ ~ 1 × 10^6^ ton (Husain 2006). More seriously, many organic dyes discharged in water contained aromatic rings, which made them highly toxic, non-biodegradable, carcinogenic or mutagenic to humans and aquatic organisms (Singh et al. 2015). Therefore, removal of various organic dyes was considered as important and urgent topic for wastewater treatment.

To date, there are various strategies including adsorption, filtration and degradation applied for organic dyes removal from contaminated water (Praveen et al. 2022). Among them, the adsorption has received widespread attention due to the unique advantages such as easy operation and high efficiency (Wu et al. 2020). Traditionally, activated carbon and adsorption resin were widely used, which could efficiently adsorb and concentrate organic dyes on their surface to decontaminate wastewater (Xiao et al. 2021; Fu et al. 2005). However, activated carbon and resins encountered the problem of high cost and low sustainability.

In recent years, bio-based adsorption materials have attracted widespread attention owing to their cheap price and good sustainability. Among biomaterials, protein was considered as a promising adsorption material due to the presence of large number of functional groups from different amino acids (Soon et al. 2022). Amino acid groups on protein surface can effectively bind toxic heavy metal ions, organic dyes and other contaminants in water. Amyloid fibrils are highly ordered β-sheet-rich proteins with characteristic cross-β conformations that could be produced by the fluttering of various types of proteins. (Ruggeri et al. 2015). To date, various animal-derived protein amyloid fibrils including β-lactoglobulin, egg white lysozyme have been prepared and applied as adsorbent for wastewater treatment (Silva et al. 2016; Bolisetty and Mezzenga 2016). Amyloid fibrils prepared from β-lactoglobulin (the major constituent of milk whey protein) were used as building blocks for the fabrication of the aerogels, which exhibited excellent adsorption capacities towards bentazone, bisphenol A and ibuprofen (Peydayesh et al. 2020). Meanwhile, amyloid fibrils from hen lysozyme were prepared and explored as efficient nano-biosorbents for removal of different organic dyes (reactive black 5, acid blue 29, and Victoria blue B) from water (Leung et al. 2015). These results proved that protein-based amyloid fibrils should be promising adsorption material for purification of organic dyes contaminated water. However, according to an overall sustainability footprint analysis, the animal-based amyloid fibrils exhibited much lower sustainability in consideration of techno-economic, environmental and social impact (Zhou et al. 2022). Therefore, it is desirable to develop plant-based amyloid fibrils for removal of organic dyes from contaminated water with a more sustainable manner.

More recently, plant-based amyloids fibrils were prepared from proteins extracted from potato, pea, soy bean, or rice, etc. (Josefsson et al. 2020, 2019; Munialo et al. 2014; Li et al. 2021). However, the plant-based amyloid fibrils have not been applied as adsorbents for organic dyes removal. Wheat flour is one of the most common food ingredients that contains 8–14% protein content (such as albumin, globulin, gliadin, and wheat gluten) (Goesaert et al. 2005). In this study, wheat flour-derived amyloid fibrils were prepared and used as the adsorbents for organic dyes removal. The amyloid fibrils were characterized in details and the adsorption performance towards typical organic dyes (Eosin Y (EY) and Congo red (CR)) were investigated. The adsorption kinetics and isotherms were also studied in detail. To the best of our knowledge, this is the first report for the application of plant-based amyloid fibrils for organic dyes removal, which would extend the application of amyloid fibrils, diversify the toolbox for water pollution management and might serve as new template to further inspire the synthesis of novel and superior amyloid fibrils with synthetic biology.

## Materials and methods

### Materials

Wheat flour was purchased from local supermarket that produced by Yuhuang Food Co. Ltd. (Shandong Province, China). C_2_H_5_OH, HCl, NaCl, KCl, MgCl_2_·6H_2_O, CuCl_2_·2H_2_O, CaCl_2·_2H_2_O, sodium dodecyl sulfate (SDS), Na_2_HPO_4_, KH_2_PO_4_, Thioflavin T (ThT), Congo red (CR), Eosin Y (EY) and all other chemicals were analytical grade, which were purchased from Sinopharm Group Co., Ltd. (Shanghai, China). Distilled water or Milli-Q Water (18 MΩ) was used for preparation of all solutions.

### Preparation of amyloid fibrils and characterization

The amyloid fibrils were prepared according to the method reported elsewhere (Hessick et al. 2022). In brief, 0.4 mL of 2% (w/v) NaCl solution were added to 0.1 g wheat flour, vortexed for 30 min, and centrifuged at 5000 rpm for 5 min, discarded the supernatant to remove albumins and globulins. Then, the pellets were collected and 0.4 mL of 70% (v/v) ethanol solution were added for every 100 mg of initial wheat flour, vortexed for 30 min, and centrifuged at 5000 rpm for 5 min, discarded the supernatant to remove gliadins. The remaining pellets were collected and 0.4 mL of SDS-PBS solution (add 0.5% (w/v) SDS to 0.05 M phosphate buffer solution, heat and stir the solution until completely homogeneous) were added, vortexed for 30 min, centrifuge at 5000 rpm for 5 min, discarded the supernatant to remove the SDS-soluble part of wheat glutenin. The remaining insoluble wheat glutenin was collected. Finally, 0.4 mL of 1 M HCl solution were added to the insoluble wheat glutenin, thoroughly mixed and incubated at 55 °C for 72 h. The obtained products were washed three times with deionized water and freeze-dried as the amyloid fibrils for the following use. The yield of amyloid fibrils was 79.5 mg/g wheat flour.

The microscopic morphology of the prepared wheat flour amyloid fibrils was characterized by TEM (JEM-2100, JEOL, Japan). The amyloid fibrils stained with ThT and observed by confocal laser scanning microscope (CLSM, Leica TCS SP5, Germany). The chemical bonds information of the amyloid fibrils was obtained by using the Fourier transform infrared spectroscopy (FTIR, Thermo Nicolet NEXUS 670, USA).

### Dye adsorption

Batch adsorption experiments towards CR and EY were conducted in a shaking incubator (30 °C, 180 rpm). For kinetic adsorption experiments, 0.5 g/L amyloid fibrils were mixed with 50 mL of dye solution in a 250-mL Erlenmeyer flask and performed at 30 °C. For the isothermal adsorption experiment, mixed 0.5 g/L amyloid fibrils with 10 mL of dye solution in a 30-mL glass bottle and perform at 30 °C. For the ion coexistence experiment, 0.5 g/L amyloid fibrils were mixed with 10 mL of dye solution (doped with 0.1 M different metal ions) in a 30-mL glass bottle and perform at 30 °C. For the recycling experiment, the amyloid fibrils were separated from the dye solution after adsorption equilibrium, the supernatant was discarded. Then the dye-adsorbed amyloid fibrils were washed several times with ethanol. The amyloid fibrils were collected by centrifugation and reused for adsorption again.

To evaluate the adsorption efficiency, the residual concentration of the dye is determined by a UV/Vis spectrophotometer (MAPADA, UV1200, China). The adsorption capacity (*Q*_e_) and adsorption ratio (*E*) are calculated using the following equations:

1 $$Q_{{\text{e}}} = \frac{{(C_{0} - C_{{\text{e}}} ) \times V}}{W},$$

2 $$E = \frac{{C_{0} - C_{{\text{e}}} }}{{C_{0} }} \times 100\% ,$$

where *C*_0_ and *C*_e_ are the initial and final concentrations of the dye in solution, and *V* (mL) is the volume of solution, *W* (mg) is the dry weight of amyloid fibers.

## Results and discussion

### Preparation and characterization of amyloid fibrils from wheat flour

**Fig. 1:**
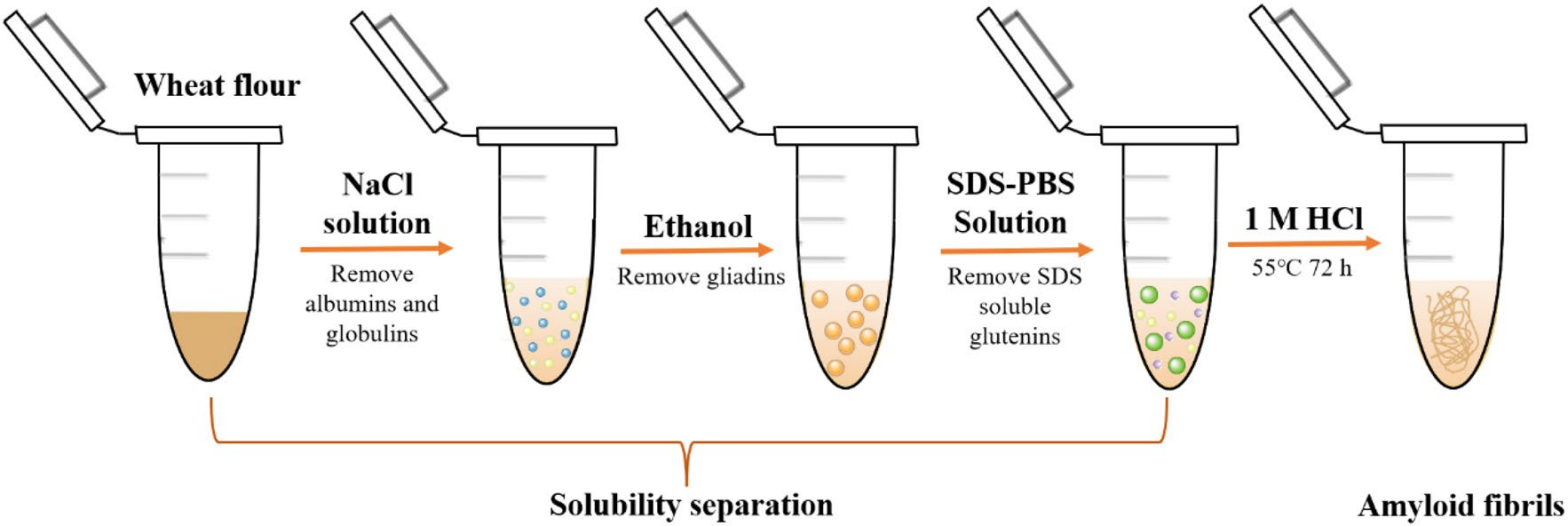
HYPERLINK "sps:id::fig1||locator::gr1||mediaobject::0"The schematic for the preparation of wheat flour amyloid fibrils

Wheat flour mainly contains albumin, globulin, gliadin, glutenin, but only the SDS-insoluble subunits of glutenin can form amyloid fibrils under acidic conditions (Goesaert et al. 2005). Therefore, albumins, globulins, gliadins were firstly removed from the wheat flour (Fig. 1). As albumins and globulins could solve in NaCl solution, these proteins were removed by dissolving the wheat flour in 2% (w/v) NaCl solution. Next, ethanol was added to dissolve and remove gliadins. After that, sodium dodecyl sulfate-phosphate buffer saline (SDS-PBS) solution was added to remove SDS-soluble glutenin subunits. Finally, the SDS-insoluble glutenin subunit was collected and washed for amyloid fibrils preparation. For amyloid fibrils formation, 1 M HCl was used to induce the fibrosis under 55 °C for 72 h (Additional file 1: Fig. S1).

**Fig. 2:**
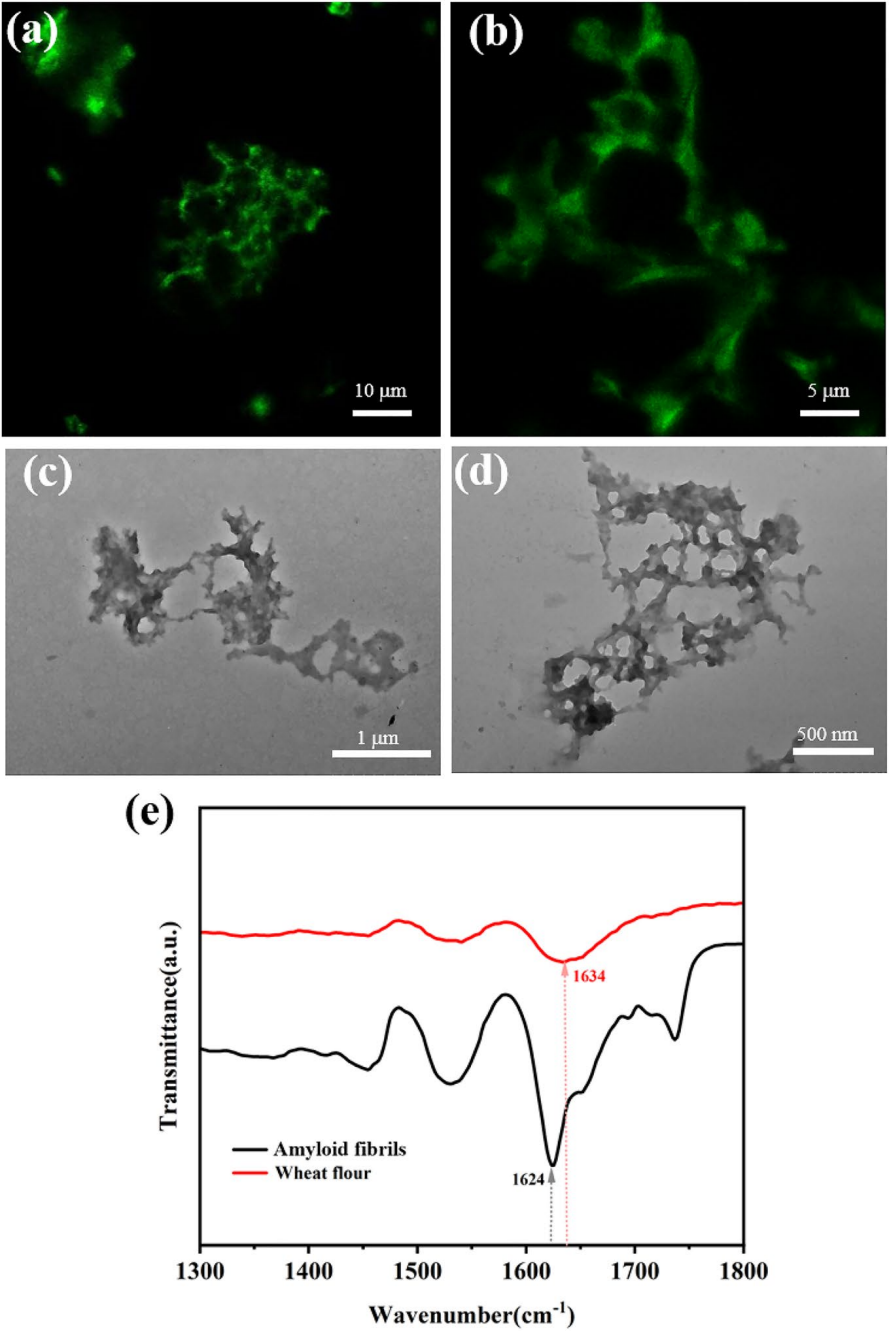
Characterization of the amyloid fibrils. **a**, **b** Confocal microscopy images of ThT-stained amyloid fibrils. **c**, **d** TEM images of amyloid fibrils. **e** FTIR spectrum of amyloid fibrils and wheat flour

To confirm the formation of wheat flour amyloid fibrils, ThT staining, TEM observation and FTIR analysis were applied. As ThT dyes can selectively bind to amyloid fibrils (Hackl et al. 2015), the amyloid products prepared from wheat flour were stained with ThT and characterized by CLSM. It was found that obvious fibrils structures stained with green fluorescence was observed with the fluorescence microscope (Fig. 2a, b). The results suggested amyloid fibrils were prepared from the wheat flour. Next, the morphology of the amyloid fibrils was observed by TEM. According to the TEM images (Fig. 2c, d), typical fibrils network that inter-connected together were formed, which confirmed the formation of amyloid fibrils. In addition, FTIR spectroscopy was used to study functional groups on the surface of amyloid fibrils. As shown in Fig. 2e, the C=O telescopic vibration absorption peak is highlighted in the spectrum region of the amide I band (1600–1700 cm^−1^). Interestingly, an obvious peak shift of amide I was observed (shifted from 1634 cm^−1^ to a lower wavenumber of 1624 cm^−1^), indicating the structural transition in solution from random coiled polypeptides to β-sheet-rich amyloid fibrils (Zhou et al. 2022). All these results confirmed that the amyloid fibrils from wheat flour was successfully prepared.

### Decolorization performance of amyloid fibrils

**Fig. 3:**
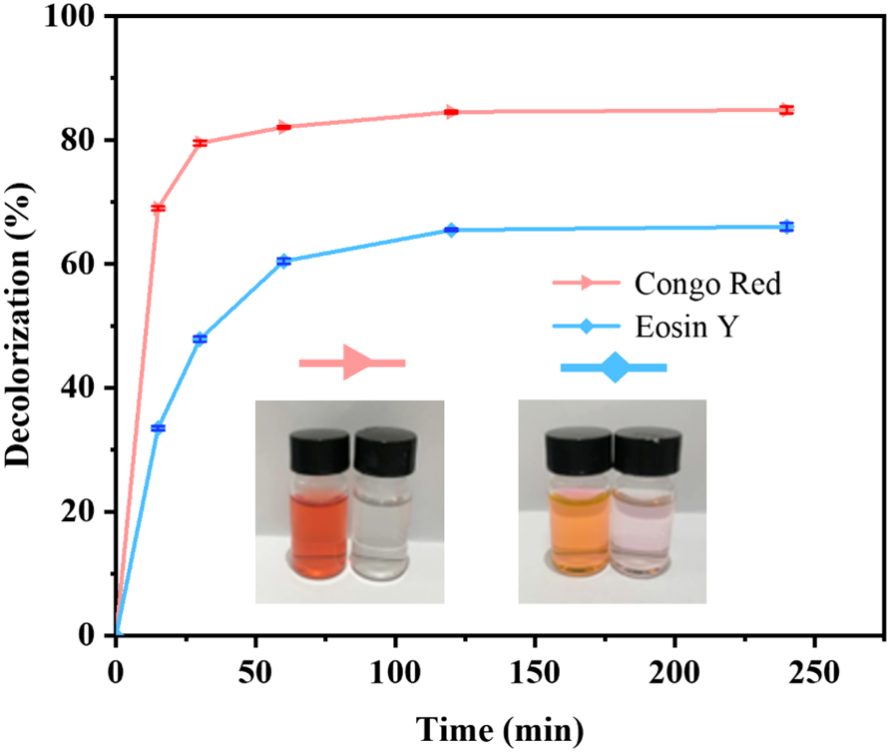
Decolorization of different dyes by amyloid fibrils. The insets are the photographs of each dye solution before (left) and after (right) amyloid fibrils treatment. Experimental conditions: adsorbent dose 0.5 g/L; initial concentration: CR (100 mg/L), EY (50 mg/L); temperature: 30 °C

To evaluate the performance of wheat flour-derived amyloid fibrils in removing organic dyes from water, CR and EY were selected as the typical organic dyes for adsorption capacity analysis. Upon the addition of amyloid fibrils, the color of CR or EY solution gradually decreased and finally turn to nearly colorless solution, suggesting good decolorization performance. As shown in Fig. 3, the dyes were quickly adsorbed to the amyloid fibrils at the first 15 min. For CR, the decolorization ratio reached about 70% in first 15 min incubation, and reach the plateau of ~ 80% when incubated with 30 min. For EY, the decolorization ratio reached about 32% in first 15 min incubation, while reached the highest adsorption of over 60% in 120 min. Full UV–vis spectrum analysis was also applied to confirm this adsorption (Additional file 1: Figs. S2 and S3). It was clear that the main adsorption peaks from CR nearly disappeared, and nearly no new adsorption peaks were observed. We also explored the effects of different metal ions on the adsorption of CR by wheat flour amyloid fibrils in Additional file 1: Fig. S5. The results showed that when 0.1 M Na^+^, Ca^2+^, Mg^2+^ and Cu^2+^ were added to CR containing 100 mg/L, there was no significant change in the adsorption capacity of CR, indicating that co-existing ions did not significantly affect the adsorption efficiency of dyes by amyloid fibrils. For EY, the main adsorption peaks were largely decreased, while also no new peaks observed. These results confirmed that CR and EY were adsorbed by amyloid fibrils isolated from wheat flour.

### Adsorption kinetics of wheat flour amyloid fibrils

**Fig. 4:**
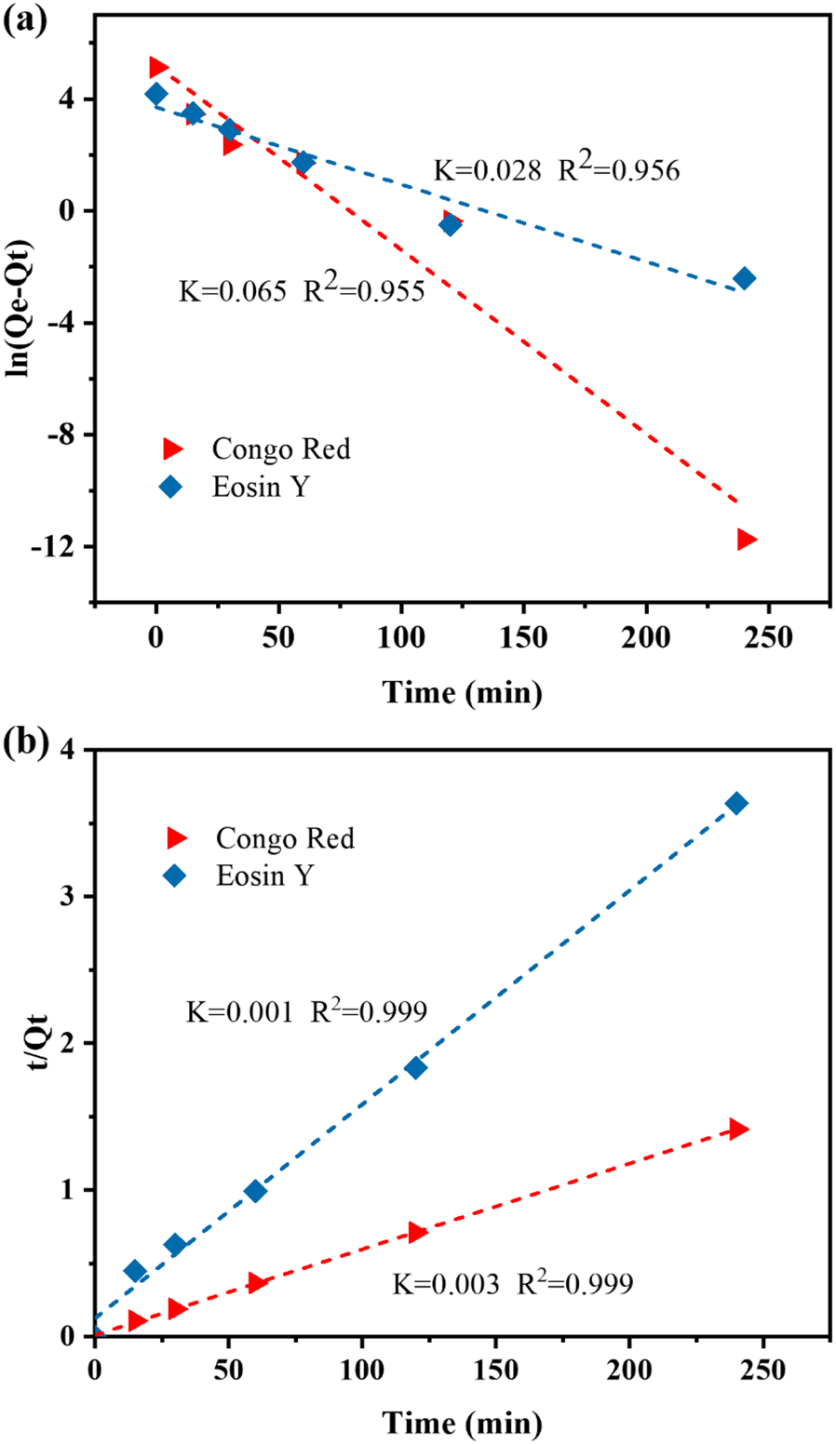
Pseudo-first-order (**a**) and pseudo-second-order (**b**) kinetics plots of dyes adsorption by the wheat flour amyloid fibrils. Experimental conditions: adsorbent dose 0.5 g/L; initial concentration: CR (100 mg/L), EY (50 mg/L); temperature: 30 °C

The adsorption kinetics were further analyzed with pseudo-first-order model (Fig. 4a) and pseudo-second-order model (Fig. 4b) with the following equations (Azizian 2006):

3 $$\ln (Q_{{\text{e}}} - Q_{t} ) = \ln Q_{{\text{e}}} - {\rm K}_{1} t\quad \left( {\text{pseudo - first - order}} \right),$$

4 $$\frac{t}{{Q_{t} }} = \frac{t}{{Q_{{\text{e}}} }} + \frac{1}{{K_{2} Q_{{\text{e}}}^{2} }}\quad \left( {\text{pseudo - first - order}} \right),$$

where *Q*_t_ (mg/g) and *Q*_e_ are the amount of adsorbed dye in the *t* (min) moment and equilibrium state (mg/g), *K*_1_ (1/min) is a pseudo-first-order rate constant, and *K*_2_ (g/mg/min) is a pseudo-second-order rate constant. The experimental adsorption processes were fitted to these two models according to Eqs. (3) and (4) and the kinetic parameters and correlation coefficient *R*^2^ were estimated (Fig. 4, Table 1). For CR and EY dyes, the correlation coefficient *R*^2^ value of the pseudo-first-order equation (the *R*^2^ value is smaller than 0.99) is smaller than the correlation coefficient *R*^2^ value of the pseudo-second-order equation (the *R*^2^ value is greater than 0.99). In details, for CR adsorption, the *R*^2^ values of the pseudo-first and pseudo-second orders were 0.955 and 0.999, respectively. For the adsorption of EY, the *R*^2^ of the pseudo-first and pseudo-second orders were 0.956 and 0.999, respectively. In addition, the difference between the experimental value (*Q*_e,exp_) and the calculated value (*Q*_e,cal_) of the equilibrium adsorption capacity of the first-order fitting was much larger than the difference between the second-order fitting. These results indicated that the adsorption by wheat flour amyloid fibrils was a pseudo-second-order process, which followed the pseudo-second-order kinetic mechanism. For CR, the experimental equilibrium adsorption capacity *Q*_e__,__exp_ was 170 mg/g, and the equilibrium adsorption capacity *Q*_e__,__cal_ calculated by the pseudo-second-order fitting was 172 mg/g. The equilibrium adsorption capacity *Q*_e__,__exp_ of EY was 66 mg/g, and the equilibrium adsorption capacity *Q*_e_ calculated by the pseudo-second-order fitting was 70.4 mg/g (Table 1).

**Table 1:** Adsorption kinetic parameters

Models	Parameters	Dyes	
		CR	EY
Pseudo-first-order	*C*_0_ (mg L^−1^)	100	50
	*K*_1_ (min^−1^)	0.065	0.028
	*Q*_e_, cal (mg g^−1^)	149	40.8
	*R*^2^	0.955	0.956
Pseudo-second-order	*K*_2_ (g mg^−1^ min^−1^)	0.003	0.001
	*Q*_e_, exp (mg g^−1^)	170	66
	*Q*_e_, cal (mg g^−1^)	172	70.4
	*R*^2^	0.999	0.999

### Adsorption isotherms of wheat flour amyloid fibrils

The experimental data for amyloid fibrils adsorption of CR and EY were fitted with the Langmuir and Freundlich isotherm model as described by the following equations (Vasanth Kumar and Sivanesan 2006):

5 $$\frac{{C_{{\text{e}}} }}{{Q_{{\text{e}}} }} = \frac{{C_{{\text{e}}} }}{{Q_{{\text{m}}} }} + \frac{1}{{K_{{\text{L}}} Q_{{\text{m}}} }}\quad \left( {{\text{Langmuir}}} \right),$$

6 $$\ln Q_{{\text{e}}} = \ln K_{{\text{F}}} + \frac{1}{n}\ln C_{{\text{e}}} \quad \left( {{\text{Freundlich}}} \right).$$

*Q*_e_ (mg/g) is the equilibrium adsorption capacity, *C*_e_ (mg/L) is the equilibrium concentration of solute, *Q*_m_ (mg/g) is the maximum monolayer adsorption capacity, *K*_L_ is the Langmuir constant related to the adsorption energy, *K*_F_ is an indicative of the relative adsorption capacity of the amyloid, and 1/*n* is the adsorption intensity.

**Fig. 5:**
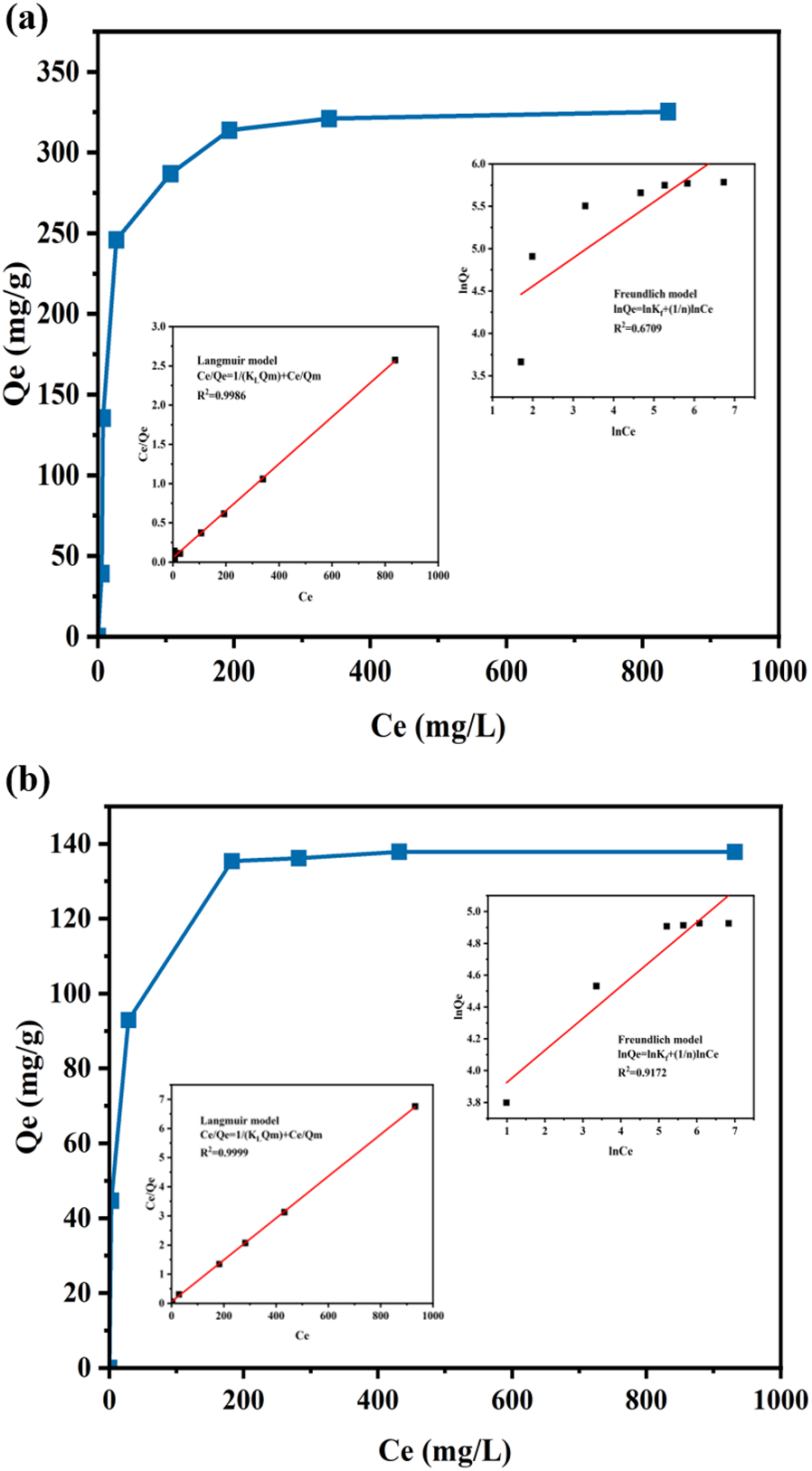
Adsorption isotherm curves for the adsorption of CR (**a**) or EY (**b**) by the wheat flour amyloid fibrils. The experimental data were simulated by Langmuir and Freundlich models (insets). Experimental conditions: adsorbent dose 0.5 g/L; temperature: 30 °C

The isothermal adsorption curve of amyloid fibrils to CR or EY was fitted with Langmuir (Eq. (5)) and Freundlich (Eq. (6)) models, respectively (Fig. 5 and Table 2). For CR, the *R*^2^ value of Langmuir fitting (0.999) was much higher than that of the *R*^2^ (0.671) from Freundlich fitting, indicating CR adsorption on amyloid fibrils fitted well with the Langmuir isotherm model. For EY, Langmuir's *R*^2^ value (0.999) was also higher than that from Freundlich (0.917) model, indicating the EY adsorption on amyloid fibrils also fitted well with the Langmuir isotherm model. The results substantiated that the adsorption of CR and EY by wheat flour amyloid fibrils was a monolayer adsorption process. The maximum monolayer adsorption capacity for CR was 333 mg/g, while it was 139 mg/g for EY (Table 2). In addition, we tested the adsorption capacity of initial wheat flour for CR and EY (Additional file 1: Fig. S4). The results showed that the adsorption capacity of amyloid fibrils for CR was 2.7 times that of initial wheat flour, and the adsorption capacity of EY was 2.2 times that of initial wheat flour, which proved that plant-derived amyloid fibrils had an adsorption advantage. According to previous reports, the maximum adsorption capacity of different animal-based amyloid fibrils was among 25–260 mg/g (Additional file 1: Table S1). Although the adsorption capacity largely varied between different organic dyes and different amyloid fibrils had different selectivity, the *Q*_m_ obtained by wheat flour amyloid fibrils were comparable to other animal-based amyloid fibrils. According to previous report, the adsorption mechanism for the amyloid fibrils might include the synergistic contributions such as electrostatic attraction, hydrophobic interaction, hydrogen bonding, π–π stacking, β-sheet affinity, and pore-filling mechanism (Liang et al. 2022). Therefore, considering the sustainability of the plant-based amyloid fibrils, the wheat flour amyloid fibrils would be promising and sustainable adsorbents for practical application. The regeneration ability of amyloid fibrils for organic dyes adsorption was also tested (Additional file 1: Fig. S6). It was shown that the removal efficiency of amyloid fibrils also reached over 90% after 3 continuous reuse cycles, indicating the amyloid fibrils could be regenerated for application.

**Table 2:** Adsorption isotherm parameters

Models	Parameters	Dyes	
		CR	EY
Langmuir	*Q*_m_ (mg g^−1^)	333	139
	*K*_L_ (L mg^−1^)	0.055	0.130
	*R*^2^	0.999	0.999
Freundlich	1/n	0.331	0.201
	*K*_F_ (mg g^−1^)/(mg L^−1^)^*n*^	49.2	41.5
	*R*^2^	0.671	0.917

## Conclusions

In summary, plant-based amyloid fibrils were successfully prepared from wheat flour by a simple and scalable method. The presence of amyloid fibrils was confirmed by ThT staining, TEM observation and FTIR analysis. Further, the wheat flour-derived amyloid fibrils exhibited excellent adsorption capacity towards organic dyes with the maximum adsorption capacity of 333 mg/g towards CR, which was superior to other reported amyloid fibrils. Adsorption kinetics and isotherms analysis indicated the amyloid fibrils adsorption process followed the pseudo-second-order kinetic and Langmuir monolayer adsorption mechanisms. This finding demonstrated the power of plant-derived amyloid fibrils for organic dyes removal from contaminated water, and provided a sustainable and scalable approach to remediate the organic dyes polluted water.

### Supplementary Information

**Additional file 1: Figure S1. **Photograph of amyloid fibrils preparation process (a. wheat flour, b. amyloid fibrils). **Figure S2.** Comparison of full spectrum of CR solution before and after amyloid fibrils adsorption. **Figure S3.** Comparison of full spectrum of full spectrum of EY solution before and after amyloid fibrils adsorption. **Figure S4.** Adsorption capacity of CR and EY by the wheat flour. **Figure S5.** Effect of different metal ions on adsorption of CR by the amyloid fibrils. **Figure S6.** Cyclic removal efficiency of CR by the wheat flour amyloid fibrils. **Table S1. **Adsorption performance of amyloid fibrils derived from natural materials.

## Data Availability

All relevant data are included in the paper or its Supplementary Information.
