# Supplementary material for: Wheat flour-derived amyloid fibrils for efficient removal of organic dyes from contaminated water
Source: Bioresour Bioprocess. 2024 Feb 14;11(1):22. doi: 10.1186/s40643-024-00737-9 (PMC10991873; doi:10.1186/s40643-024-00737-9)
Supplement: Supplementary file 1 — Additional file 1: Figure S1. Photograph of amyloid fibrils preparation process (a. wheat flour, b. amyloid fibrils). Figure S2. Comparison of full spectrum of CR solution before and after amyloid fibrils adsorption. Figure S3. Comparison of full spectrum of full spectrum of EY solution before and after amyloid fibrils adsorption. Figure S4. Adsorption capacity of CR and EY by the wheat flour. Figure S5. Effect of different metal ions on adsorption of CR by the amyloid fibrils. Figure S6. Cyclic removal efficiency of CR by the wheat flour amyloid fibrils. Table S1. Adsorption performance of amyloid fibrils derived from natural materials. [file 40643_2024_737_MOESM1_ESM.docx]

**Additionaly Information**

**Wheat flour-derived amyloid** **fibrils for efficient removal of organic dyes from contaminated water**

Dan-Dan Yang^1^, Fu-Xiang Chang^1,2^, Bo-Fan Zhang^1,2^, Yang-Chun Yong^1,2,3,*^

*^1^Biofuels Institute, School of Environment and Safety Engineering, Jiangsu University, 301 Xuefu Road, Zhenjiang 212013, China*

*^2^School of Emergency Management, Jiangsu University, 301 Xuefu Road, Zhenjiang 212013, China*

*^3^Jiangsu Collaborative Innovation Center of Technology and Material of Water Treatment, Suzhou University of Science and Technology, Suzhou 215009, China*

*Corresponding author, Email: ycyong@ujs.edu.cn; Fax: +86-511-8879 0931


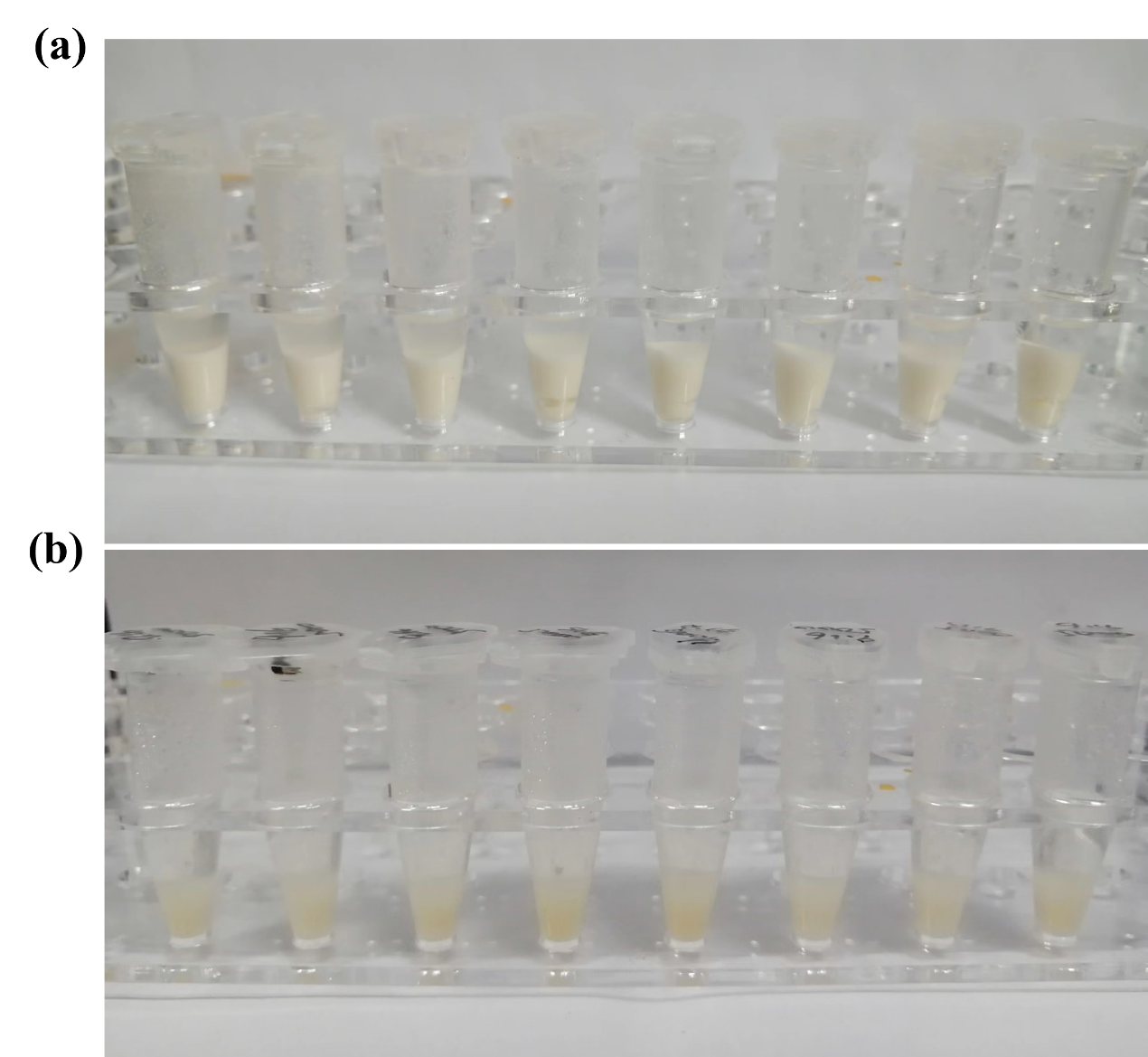


**Figure S1.** Photograph of amyloid fibrils preparation process (a. wheat flour, b. amyloid fibrils).


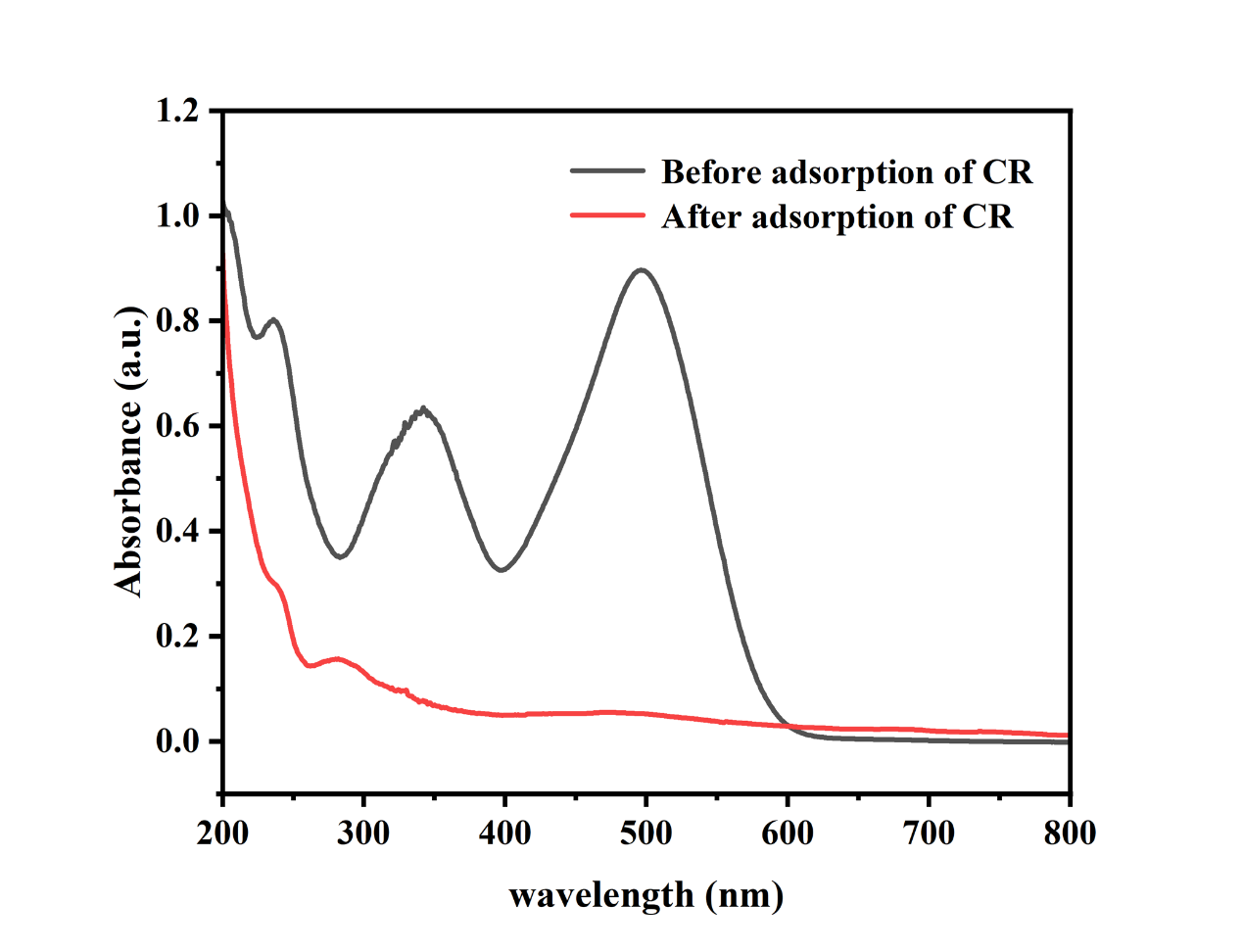


**Figure S2.** Comparison of full spectrum of CR solution before and after amyloid fibrils adsorption.


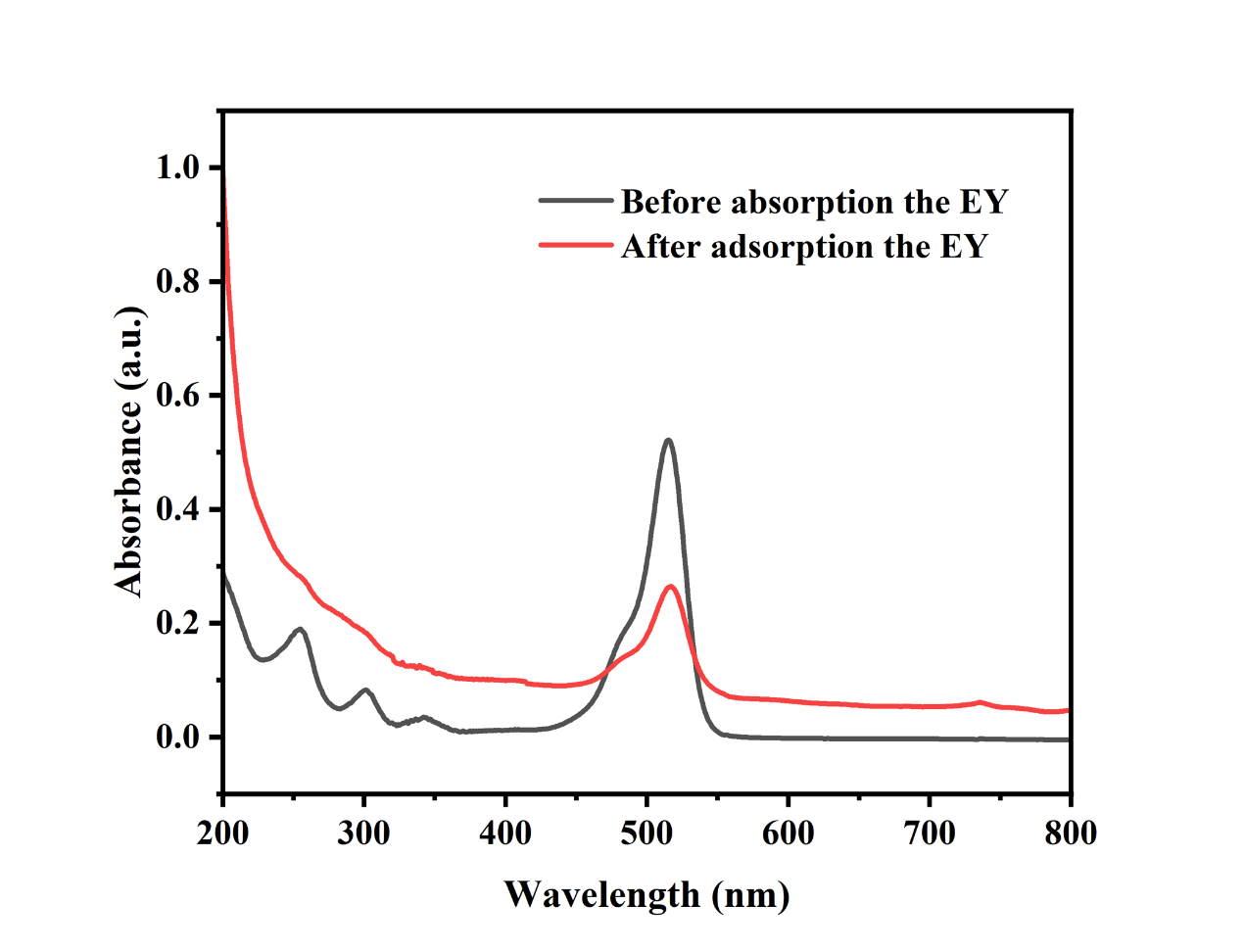


**Figure S3.** Comparison of full spectrum of full spectrum of EY solution before and after amyloid fibrils adsorption.

**
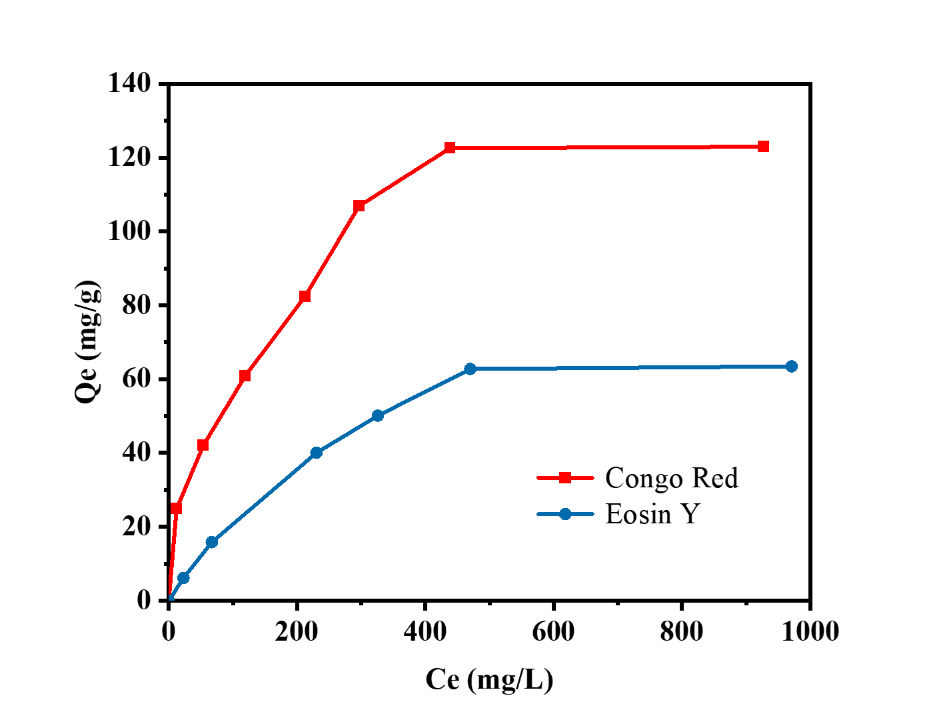
**

**Figure S4.** Adsorption capacity of CR and EY by the wheat flour.

**
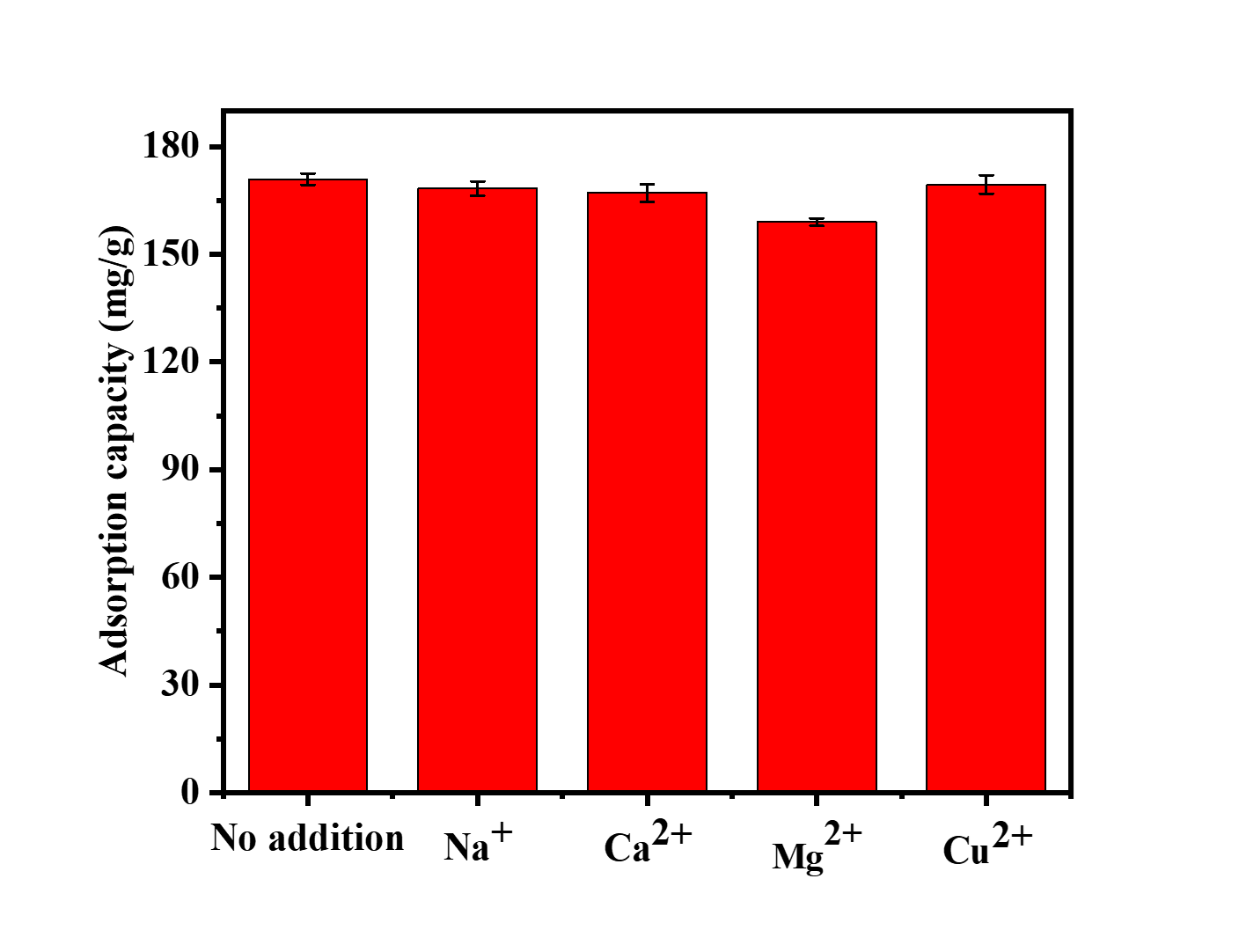
**

**Figure S5.** Effect of different metal ions on adsorption of CR by the amyloid fibrils.

**
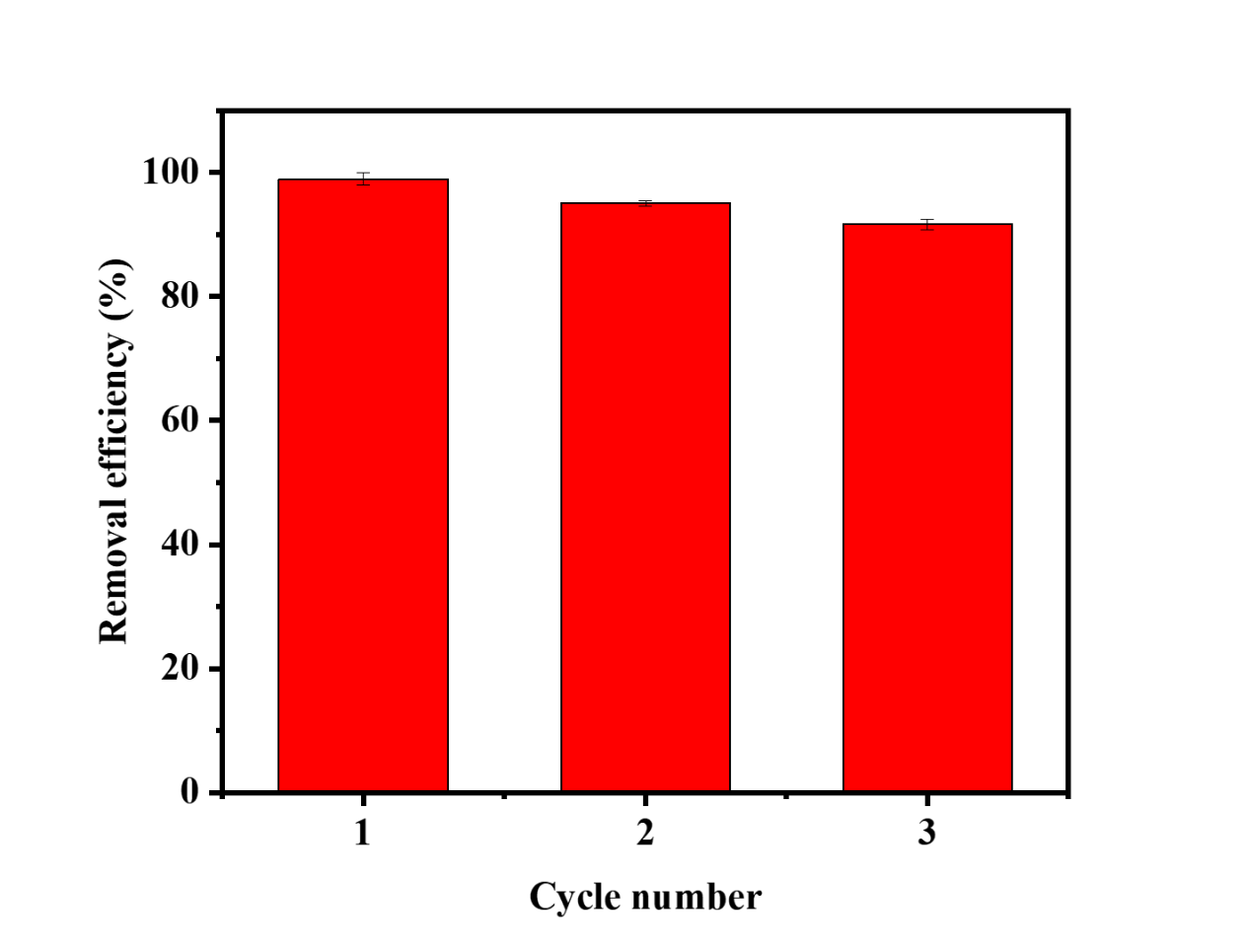
**

**Figure S6.** Cyclic removal efficiency of CR by the wheat flour amyloid fibrils.

**Table S1.** Adsorption performance of amyloid fibrils derived from natural materials

| Amyloid | Source | Dyes | Qe(mg/g) | Qm(mg/g) | Reference |
| --- | --- | --- | --- | --- | --- |
| Amyloid fibrils of hen lysozyme | Egg white | Reactive Black 5 | 155 | 159 |  |
|  |  | Acid Blue 29 | 101 | 103 | (Leung et al. 2015) |
|  |  | Victoria Blue B | 86 | 100 |  |
| β-lactoglobulin amyloid fibril/UiO-66-NH_2_ | Milk | Rhodamine B | 54.8 |  |  |
|  |  | Crystal violet | 27.1 |  | (Peydayesh et al. 2022a) |
|  |  | Methylene blue | 25.2 |  |  |
|  |  | Malachite green | 29.6 |  |  |
| amyloid whey lactose carbon aerogel | Milk | Rhodamine B | 125 |  | (Peydayesh et al. 2022b) |
| carboxymethylated cellulose nanofibril (CNF) and β-lactoglobulin amyloid nanofibril (ANF) based aerogel-like adsorbents | milk | Brilliant blue |  | 67.9 | (Atoufi et al. 2022) |
| Amyloid fibrils from wheat flour protein | Wheat flour | Congo red | 170 | 333 | This study |
|  |  | Eosin Y | 66 | 139 |  |

**Reference：**

Atoufi Z, Cinar Ciftci G, Reid MS, Larsson PA, Wågberg, L (2022) Green Ambient-Dried Aerogels with a Facile pH-Tunable Surface Charge for Adsorption of Cationic and Anionic Contaminants with High Selectivity. Biomacromolecules 23(11): 4934-4947. <https://doi.org/10.1021/acs.biomac.2c01142>

Leung WH, Lo WH, Chan PH (2015) Amyloid fibrils as rapid and efficient nano-biosorbents for removal of dye pollutants. RSC Adv 5(109): 90022-90030. https://doi.org/[10.1039/c5ra17182b](https://www.x-mol.com/paperRedirect/50493)

Peydayesh M, Chen X, Vogt J, Donat F, Müller R, Mezzenga R (2022a) Amyloid fibril-UiO-66-NH_2_ aerogels for environmental remediatio. Chem Comm 58(33): 5104-5107. https://doi.org/[10.1039/d2cc00695b](https://www.x-mol.com/paperRedirect/1509607008512303104)

Peydayesh M, Vogt J, Chen X, Zhou J, Donat F, Bagnani M et al (2022b) Amyloid-based carbon aerogels for water purification. Chem Eng J 449: 137703. <https://doi.org/10.1016/j.cej.2022.137703>
